# Impact of a community health worker led intervention for improved blood pressure control in urban Nepal: an open-label cluster randomised controlled trial

**DOI:** 10.1016/j.lansea.2024.100461

**Published:** 2024-08-08

**Authors:** Sanju Bhattarai, Eva Skovlund, Archana Shrestha, Bente Prytz Mjølstad, Bjørn Olav Åsvold, Abhijit Sen

**Affiliations:** a Department of Public Health and Nursing, Faculty of Medicine and Health Sciences, Norwegian University of Science and Technology, Trondheim, Norway; b Institute for Implementation Science and Health, Kathmandu, Nepal; c Department of Public Health, Kathmandu University School of Medical Sciences, Dhulikhel, Nepal; d Department of Chronic Disease Epidemiology, Center of Methods for Implementation and Prevention Science, Yale School of Public Health, New Haven, USA; e General Practice Research Unit, Department of Public Health and Nursing, Faculty of Medicine and Health Sciences, Norwegian University of Science and Technology, Trondheim, Norway; f Department of Endocrinology, Clinic of Medicine, St. Olavs Hospital, Trondheim University Hospital, Trondheim, Norway; g Center for Oral Health Services and Research (TkMidt), Trondheim, Norway

**Keywords:** Hypertension, Public health, Nepal, Comprehensive intervention, Community health workers

## Abstract

**Background:**

Effective control of hypertension remains challenging in low and middle-income countries. We tested the effectiveness of comprehensive approaches to hypertension management including six home visits by community health workers with regular follow up by a trained healthcare provider on blood pressure levels in Nepal.

**Methods:**

We implemented a non-blinded, open-label, parallel-group, two-arm cluster randomised controlled trial, with 1:1 allocation ratio in Budhanilakantha municipality, Kathmandu, Nepal. Ten public health facilities and their catchment area were randomly allocated to receive comprehensive intervention or only usual hypertension care. We recruited 1252 individuals aged 18 years and older with hypertension. The primary outcome was systolic blood pressure. Secondary outcomes were diastolic blood pressure, proportion with controlled blood pressure, waist to hip ratio, body mass index, physical activity, diet quality score, daily salt intake, adherence to antihypertensives, hypertension knowledge and perceived social support. Primary analysis was by intention-to-treat using a linear mixed model.

**Findings:**

Participants were, on average 57 years old, 60% females, 84% married, 54% Brahmin/Chettri ethnicity and 33% were illiterate. The decrease in mean systolic blood pressure (1.7 mm Hg, 95% CI −0.1, 3.4) and diastolic blood pressure (1.6 mm Hg, 95% CI 0.5, 2.6) was more in the intervention arm compared to the control. The proportion with blood pressure control (OR 1.5 95% CI 1.0, 2.1) and engaging in adequate physical activity (≥600 Metabolic equivalents of task per week) (OR 2.2, 95% CI 1.6, 3.1) were higher in the intervention arm compared to control. The change in hypertension knowledge score was higher and daily salt intake was lower in the intervention arm compared to control. Waist to hip ratio increased more and global dietary requirement scores decreased more in the intervention group and there was no effect on the body mass index and adherence to antihypertensives.

**Interpretation:**

Community health workers facilitated home support and routine follow-up care by healthcare providers was effective in controlling blood pressure in urban Nepal. These findings suggest comprehensive interventions targeting individual, community and health system barriers are feasible in low resource settings, but larger implementation trials are needed to inform future scale-up.

**Funding:**

This work was supported by 10.13039/100009123Norwegian University of Science and Technology, Trondheim, Norway (Project number 981023100).

**Research in context**

**Evidence before this study**

Effective control of hypertension remains challenging in low and lower middle-income countries (LLMICs). A 2018 review by Mills and colleagues found that comprehensive (multicomponent) interventions were most effective in controlling blood pressure among individuals with hypertension. They also highlighted paucity of implementation studies from LLMICs on blood pressure reduction. In 2024, we updated this review to include studies published between October 2017, and December 2023 in MEDLINE and EMBASE. We restricted our search to completed clinical trials in LLMICs and excluded studies solely evaluating effectiveness of drugs. Our search yielded 301 publications, of which 11 articles were eligible for inclusion (refer Supplementary Tables S1–S3 for search terms, inclusion criteria and results). We reviewed 14 articles combining with 3 LLMICs articles from the Mills and colleagues 2018 review.

All except one hypertension interventions provided some form of behaviour change strategies aiming to improve knowledge and self-efficacy for hypertension management. Overall, hypertension trials from LLMICs with 6–24 months follow-up period reduced systolic blood pressure (range between −0.31 and 21 mm Hg). However, only two trials implemented a comprehensive intervention including home support by CHWs, together with training of physicians and none were from Nepal. In summary, this review reveals lack of implementation and evaluation of comprehensive hypertension interventions addressing patient, family, and health system barriers from Nepal.

**Added value of this study**

We implemented a comprehensive intervention using community health workers (CHWs) to visit and engage hypertension patient and family members at home in developing and implementing actions to support home blood pressure monitoring, lifestyle modification, and adherence to antihypertensives while encouraging to visit trained public healthcare providers for routine follow-up care for hypertension management. To our knowledge, our study is the first to test the combined effect of these approaches in Nepal. We show that comprehensive intervention tends to reduce systolic and diastolic blood pressure and dietary salt intake and improve physical activity. However, we find an increase in waist to hip ratio and decrease in diet quality while there is no effect on body mass index and adherence to antihypertensives.

**Implications of all the available evidence**

Our trial results support existing literature showing that comprehensive intervention using CHW can control blood pressure and reduce blood pressure, though the reduction in systolic blood pressure was modest. The modest results may have been due to a short intervention period (6 months) to optimise the advantage from using a dialogical approach in solving hypertension management problems. Blood pressure control continues to be an intractable problem and innovation in the control of blood pressure is an urgent priority. Future efforts could therefore consider testing comprehensive (multicomponent) interventions, such as those tested in our trial, for longer duration and within larger scale community programs to increase impact.

## Introduction

Effective control of blood pressure remains unattainable in low and middle-income countries (LMICs).^1^ Deaths from cardiovascular diseases (CVDs) attributed by hypertension occur more frequently in LMICs than in high income countries^1^ and none of the LMICs are on track to meet the global target of reducing CVD mortality by 30% by 2030.^2^ Although antihypertensives are available and can effectively reduce high blood pressure and the onset of its complications^3^ countries are struggling to identify individuals, initiate treatment, and control hypertension.^1^ In Nepal in 2019, 11% of individuals aware of hypertension were on treatment, which is almost unchanged from the 2013 estimate of 10%.^4^

Despite Nepal's attempts to manage hypertension through early diagnosis and treatment its effective control is alarmingly low.^5^ Proven cost-effective therapeutic treatments for CVDs often fail to reach those most in need,^6^^,^^7^ and health system challenges such as poor patient counselling and long waiting times hinder access to hypertension care and treatment.^8^, ^9^, ^10^, ^11^, ^12^ Community health workers (CHWs) facilitated home visits have been used to overcome some of these barriers by improving access to counselling, reducing the frequency of hospital visits, and engaging patient and family members in home management of hypertension. Compared to standard care, CHWs led blood pressure monitoring and counselling intervention had greater reduction in systolic blood pressure in hypertension patients in South Asia (Bangladesh, Pakistan, and Sri Lanka).^13^ A systematic review largely including studies from high-income countries has shown the benefit of implementing multicomponent strategies for longer than six months on hypertension control.^14^ Interventions to provide personalised counselling through health volunteers in rural Nepal have successfully reduced average blood pressure,^15^ although the results were not sustained over time.^16^ However, the effect of using CHWs (nurses and health assistants who have three years of medical training as physician's assistants) for personalised home-based support to overcome barriers in controlling high blood pressure, medication adherence and adoption of healthy behaviours has not been previously tested in Nepal. We aimed to fill this research gap by conducting a cluster randomised controlled trial testing the effectiveness of a ‘comprehensive approach to hypertension management’ intervention. The intervention consisted of home visits by CHWs to engage hypertension patients and family members to self-monitor blood pressure, provide counselling tailored to individual and family context and referring patient for regular follow-up with trial trained primary health care providers to audit blood pressure logs and reassess treatment plan in Budhanilakantha municipality, Kathmandu, Nepal. Our intervention package was informed by a formative study assessing contextual facilitators and barriers in managing hypertension, whose findings are already published.^17^ We hypothesised that providing regular monthly home visits would decrease blood pressure, compared with usual care. The primary objective was to assess whether the contextualised comprehensive intervention, in addition to usual care, decreased blood pressure of individuals with hypertension compared with those who only had access to usual care. Further, we assessed impacts on control of blood pressure, diet, physical activity, and knowledge of hypertension.

## Methods

### Trial design

The trial was an open-label, non-blinded parallel group two arm cluster randomised superiority trial. In the control arm hypertension patients had access to usual hypertension care. The detailed methodology is previously published and no important changes were made in the methods from the published trial protocol^18^ and trial registration (https://clinicaltrials.gov/ct2/show/NCT05292469?cntry=NP&draw=2&rank=6).

### Study population and settings

The trial was implemented in Budhanilakantha, an urban municipality in Bagmati province, Nepal. The municipality has 11 primary care public health facilities including one municipal hospital. According to the 2023 census,^19^ the municipality population is 177,577 with a sex ratio of 96.8 males per 100 females, 70% older than 20 years, and 71% of the population having migrated from other parts of the country. The literacy rates of men and women are 96% and 85% respectively. The predominant ethnicity was Brahmin/Chettri. In Bagmati province nearly 53% of all reported deaths were from non-communicable diseases.^19^

### Randomisation and masking

The primary public healthcare facilities were randomly assigned with 1:1 allocation to receive the comprehensive intervention package or usual care (control arm) only. The municipal hospital was excluded as physicians provided hypertension care unlike other facilities. Trial statistician used Stata v18 to generate 10 different combinations of allocation sequences and concealed it in 10 opaque sealed envelopes. During a participatory randomisation event held at the municipality, a stakeholder picked one of the sealed envelopes, opened, and disclosed allocation to everyone present. It was not possible to blind the intervention to enumerators and participants after disclosing allocation of clusters.

### Participant recruitment and consent

After randomisation, trained enumerators (nursing or bachelor's degree in health sciences) identified participants with support from health care providers, female community health volunteers, local volunteers and during health camps. Participants aged 18 years and older, with an established hypertension diagnosis (systolic blood pressure ≥140 mm Hg and/or diastolic blood pressure ≥90 mm Hg or prescribed antihypertensives), residing in the catchment areas of the primary public health facilities (clusters), able to respond to questions, and not planning to change residence during follow-up (12 months) were eligible. Participants with high blood pressure measurement in two assessments and no prior hypertension diagnosis were referred to physician for confirmation before enrolment. Pregnant women and individuals with self-reported serious advanced illness such as terminal cancer and paralysis were excluded. Enumerators confirmed eligibility and took written consent from participants explaining the objective of the study and their role. The consent form in the intervention arm also included consent to participate in the home visits.

### Comprehensive intervention package

Two healthcare providers from each intervention public health facility received a 4 days training on package for essential non communicable diseases (PEN) for service providers.^20^ The healthcare providers audited the blood pressure logs kept by participants and followed standard protocol for referral and treatment. The participants were provided with a blood pressure monitoring device, a pillbox, and an informational package with tools to support hypertension management. Enrolled participants were assigned to one of the five CHWs who were nurses, or paramedics. A home visits manual was developed using resources from ‘Your heart, your life: A CHW's manual for the Hispanic community’.^21^ The CHWs received PEN training along with healthcare providers and received an additional 4-day training on how to use the home visit manual. Trained CHWs scheduled and visited the homes of participants enrolled in the intervention arm to offer six home visit sessions with one month gap between each visit. They filled and submitted a paper-based form to the intervention coordinator after each session. In the first visit CHWs supported hypertension patients and their families to take actions to improve blood pressure by monitoring blood pressure, adhering to medication and healthy behaviours and going for routine follow-up care. The CHWs used dialogue to trigger reflection and action supporting them to think critically about the ways to control hypertension.^22^ At the end of the first session, the participants made action plans to address the relevant issues for their family. In the subsequent session the action plans were reviewed, discussed, and updated as required. Participants were free to seek concomitant care for hypertension irrespective of allocation. Details on the intervention are described in the published protocol.^18^

### Trial surveillance

Enumerators used electronic questionnaires with in-built jump-sequences and value limits developed in KOBO toolbox to collect data. Baseline data were collected at enrollment. Socioeconomic information such as age, sex, marital status, education, occupation, family size and annual household income were self-reported. Per-capita income was calculated dividing household income by total members in the family. Comorbidity with diabetes was assessed by asking participants if they had been diagnosed with diabetes by a physician. Enumerators followed standard procedures to ensure that recall of self-reported data were accurate and inter-observer difference minimised. They received PEN training that includes sessions on anthropometric and blood pressure measurements to ensure standardised measurements. Blood pressure was measured using an Omron Hem-8712 automatic digital monitor. The mean of the last two of the three readings were used. Participants were asked to remove heavy clothing such as jackets and sweaters before anthropometric measurements. Weight in kilograms was measured using an Omron digital weighing scale. A non-elastic retractable measuring tape was used for height, and waist and hip circumference. Participants were asked to stand barefoot beside a wall on even floor to measure height. Hip circumference was measured by placing the tape parallel to the floor at the widest part of the buttocks and waist circumference by placing the tape horizontally passing it along the umbilicus as an estimate of midpoint between the 12th rib and the iliac crest on the mid axillary line. Diet quality questionnaire assessed 32 different food groups participants ate in the past 24 h.^23^ The global dietary recommendation score (ranging from 0 to 18, higher score means better diet quality) measures adherence to a healthy diet protective against non-communicable diseases and was calculated by subtracting foods recommended to limit from foods recommended as healthy and adding nine.^23^ Fruits and vegetable score ranged from 0 to 6, with scores of <3 indicating less than 400 g of fruits and vegetables.^23^ Daily dietary salt intake (grams) was self-reported and adherence to antihypertensive medication assessed using Morisky medication adherence scale-8 (MMAS scores <6 as poor adherence).^24^ A global physical activity questionnaire that measures frequency, duration and intensity (moderate and vigorous) of physical activity at work, during travel, and recreation was used to calculate metabolic equivalents of task (MET) minutes per week, the ratio of metabolic energy spent when body is moving relative to when body is resting.^25^ Hypertension knowledge score was calculated using 21 item knowledge questions with each correct answer scored as 1 and incorrect 0.^26^ Perceived social support scale was used to measure the adequacy of support participants received from family, friends, and significant others.^27^ Standard drinks per week were calculated by asking current alcohol drinkers about drinking frequency and amount of different types of alcohol^4^ and categorising into non-drinkers (≤1 standard drink per month), moderate drinkers (<3 standard drinks per week), and high drinkers (≥3 standard drinks per week). Tobacco use including both smoked and chewed was categorised as never, former, and current users. At 10–12 months from baseline all except socioeconomic and demographic data were collected again following the same procedures. CHWs and enumerators recorded adverse events such as death and reported to SB.

### Outcomes

The primary outcome was systolic blood pressure. Secondary outcomes were measured or were derived from multiple questions to generate a score or a binary indicator. Secondary outcomes included diastolic blood pressure, control of blood pressure (systolic <140 mm Hg and diastolic <90 mm Hg), hip (cms), and waist circumference (cms), body mass index (<25 kg/m^2^ and ≥25 kg/m^2^),^28^ waist to hip ratio (<1 and ≥ 1), global dietary recommendation score (0–18), self-reported daily dietary salt intake (grams), adherence to antihypertensive medication (MMAS scores <6 as poor adherence) and physical activity (<600 METs/≥600), and hypertension knowledge score (0–21) and perceived social support scale with cut off at the median (low/high).

### Statistical analysis

Our target sample size was 1250 participants, 625 in each arm, assuming at least 500 participants followed up in each arm (80% follow-up rate). Assuming an intra cluster coefficient of 0.01^29^ and a standard deviation of 14 (based on unpublished results from Dhulikhel heart study) the power to detect 3.5 mm Hg mean difference in systolic blood pressure between the arms at 12 months follow up would be 80%. Within 10 clusters (5 in each arm) participants were recruited proportionate to the total population in the catchment area of the public primary health facilities. The statistical analysis approach used will enhance power as it uses baseline measurement of all participants.^30^

We followed the statistical analysis plan in the published protocol^18^ with two exceptions. Salt intake was used in grams of salt consumed daily instead of proposed categories (<5 g/≥5 g) as almost all participants consumed more than 5 g salt daily. Furthermore, the multidimensional scale for perceived social support (MSPSS) was dichotomized at median as the scale was skewed to right. Our primary analysis followed the intention-to-treat principle. Outcomes measured at participant level (not cluster means) were analysed using a mixed model accounting for cluster design. We report 95% confidence intervals and p values from two-sided statistical tests with a 5% significance level. Correction for small number of clusters was not applied. The primary outcome was analysed using a linear mixed model with an unstructured covariance. The model included systolic blood pressure (outcome) measured at follow-up and baseline as the dependent variable, a time variable (indicating baseline as 0 and follow-up as 1) and an interaction term between time and group (arm assignment)^30^ and the random effect for cluster and individual. The coefficient of the interaction term was reported for group difference. Imputation of missing values was not done because the fitted model provides valid estimates when data were missing at random.^30^ Analysis of the secondary outcomes followed the same approach, when outcomes were binary linear mixed models with a logit link were used leading to odds ratios and 95% CI.

Sensitivity analyses were performed adjusting for preidentified covariates age, sex, marital status, education, and income. A per-protocol analysis was also carried out excluding participants receiving less than 5 home visits; and those with no follow-up assessment. Subgroup analyses were performed by adding the interaction term between randomised group and the subgroup variable into the regression model. For the primary outcome (i.e. systolic blood pressure) subgroup analyses were done by sex, body mass index (<25 kg/m^2^ and ≥25 kg/m^2^), waist to hip ratio (<1 and ≥ 1) and per-capita income (cutoff at median) at baseline. We also did an exploratory post-hoc subgroup analysis considering diabetes status (yes/no), poor blood pressure control (systolic ≥160 mm Hg or diastolic ≥100 mm Hg) and age (<60 years and ≥60 years) at baseline. All analyses were conducted using Stata 18. No interim analyses were planned or conducted. The funders had no role in the study design, data collection, data analysis, interpretation, or writing of the report.

## Results

**Fig. 1:**
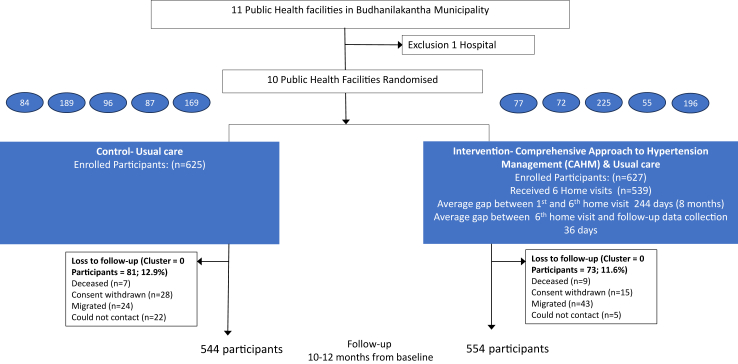
**Participants flow diagram. The figures in blue oval represents the number recruited within each cluster**.

Participants were enrolled from 2 May through 7 November 2022. The home visits took place from 17 July 2022 to 17 August 2023 and follow-up took place between 5 March and 29 August 2023. Participant flow (CONSORT diagram) is illustrated in Fig. 1. Out of 1257 eligible participants, five declined, stating they did not need the intervention and/or had a physician in the family. A total of 1252 participants were enrolled: 627 in the intervention and 625 in the control arm. Systolic blood pressure was measured for everyone at baseline and for 544 control and 554 intervention participants at follow-up.

**Fig. 2:**
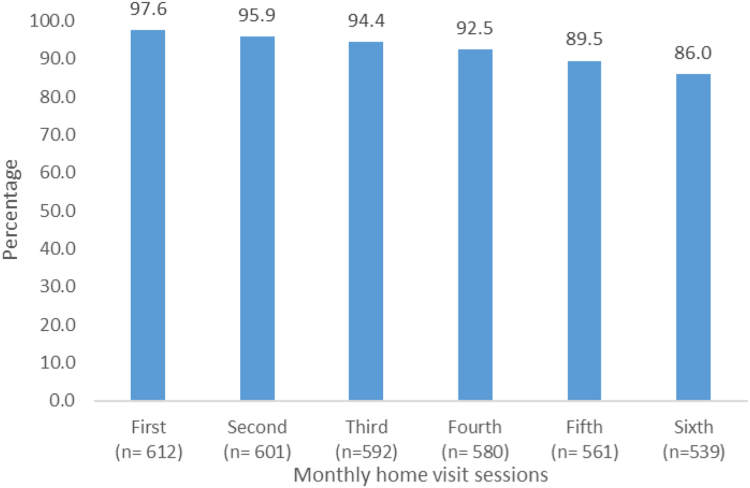
**Proportion of 627 participants in the intervention arm receiving home visit sessions**.

Loss to follow-up was 81 (13.0%) in the control arm and 73 (12.0%) in the intervention arm. Reasons were mostly due to migration, withdrawal of consent, inability to track and a small number of deaths. Characteristics of those lost to follow-up versus those retained by study arm are provided in Supplementary Table S4. There was no notable difference in loss to follow-up between the arms. Implementation mostly went as specified in the published protocol.^18^ On average it took 8 months (244 days) to complete 6 home visits which was 2 months longer than the planned 6 months. The longer duration was due to difficulty in scheduling. The average gap between completion of 6th home visit and follow-up data collection was 36 days. Adherence to home visits was high, 86% of participants received all 6 sessions (Fig. 2). Most participants (83%) recalled making action plans to reduce blood pressure. The most common actions were reducing body weight and dietary salt intake and engaging in physical activity. Two thirds of the participants followed their action plans, 28% fully and 39% partially. The most common reasons for not following agreed action plans were forgetting, often due to festivals and household work and a few due to poor family support (4%).

Baseline socio-demographic characteristics are summarised by arm in Table 1 and were mostly balanced between arms. Participants were on average 57 years old, were mostly female, had a median family size of four, were largely married, and of Brahmin/Chettri ethnicity. Around one third were illiterate and less than a third had secondary or higher education. A large proportion was unemployed (60% in intervention and 50% in control) and the median annual per capita income in US dollars was 692.3 (IQR 769.2) in intervention and 923.1 (IQR 865.4) in control arm.

**Table 1:** Socio-demographic characteristics of enrolled participants by trial arm at baseline.

Characteristics	Intervention (n = 627)		Control (n = 625)		Total (N = 1252)	
	Frequency	%	Frequency	%	Frequency	%
**Age**						
Mean, SD^a^	57.7	12.1	57.3	12.1	57.5	12.1
21–44 years	84	13.4	100	16.0	184	14.7
45–59 years	270	43.1	257	41.1	527	42.1
≥60 years	273	43.5	268	42.9	541	43.2
**Education**						
Years of education, Median, IQR^b^	2	10	3	10	3	10
Illiterate	212	33.8	201	32.2	413	33.0
Primary (0–4 grade)	213	34.0	245	39.2	458	36.6
Secondary (5–10 grade)	118	18.8	102	16.3	220	17.6
High school and above	84	13.4	77	12.3	84	12.8
**Family size**						
Median, IQR	4	3	4	2	4	3
**Female**	368	58.7	384	61.4	752	60.1
**Currently married**	531	84.7	519	83.0	1050	83.9
**Ethnicity**						
Brahmin/Chettri	326	52.0	348	55.7	674	53.8
Newar	139	22.2	99	15.8	238	19.0
Tamang/Rai/Sherpa/Magar/Gurung	118	18.8	142	22.7	260	20.8
Dalits	44	7.0	36	5.8	89	6.4
**Occupation**						
Unemployed	376	60.0	315	50.4	691	55.2
Retired	52	8.3	80	12.8	132	10.5
Paid employment	54	8.6	52	8.3	106	8.5
Self employed	145	23.1	178	28.5	323	23.1
**Per-capita annual income (USD**^c^**)**						
Median, IQR	692.3	769.2	923.1	865.4	769.2	825.6
*Income tertiles*						
Low (<577 USD)	270	43.1	162	25.9	432	34.5
Medium (578–1150 USD)	182	29.0	221	35.4	403	32.2
High (>1150 USD)	175	27.9	242	38.7	417	33.3

a Standard Deviation.

b Interquartile range.

c USD, United States Dollars (Exchange rate 1USD = 130 Nepali rupees).

One fifth of the trial participants were current smokers (Table 2). Nearly 20.8% in intervention and 22.3% in control arm were diabetic and 16.8% in intervention and 11.5% in control arm consumed 3 or more standard drinks per week. Participants were generally overweight and consumed a high salt diet. The blood pressure measurements at baseline were also balanced between the arms. Participants had a mean systolic and diastolic blood pressure 133.8 mm Hg and 86.8 mm Hg respectively. Median years since hypertension diagnosis was nearly 6.4 (IQR 8.8) and almost all were prescribed antihypertensives. They had moderate levels of hypertension knowledge, perceived to receive high social support, and adhered to antihypertensives. A larger proportion in the control arm went to a private facility for routine hypertension services than in the intervention arm.

**Table 2:** Clinical characteristics of participants by trial arm at baseline.

Characteristics	Intervention (n = 627)		Control (n = 625)		Total (N = 1252)	
	Frequency	%	Frequency	%	Frequency	%
**Current tobacco users**	131	20.9	130	20.8	261	20.9
**Standard alcoholic drinks**						
Non-drinkers	476	61.7	527	84.3	1003	80.1
<3 drinks per week	46	7.3	26	4.2	72	5.8
≥3 drinks per week	105	16.8	72	11.5	105	14.1
**Diabetic**	130	20.8	140	22.3	270	21.6
**Number of antihypertensives prescribed**						
0	30	4.8	52	8.3	87	6.9
1	307	49.0	291	46.5	598	45.6
2	247	39.4	251	40.2	498	39.6
3–4	43	6.8	31	5.0	74	5.9
**Type of health facility for seeking hypertension care**						
Public	163	26.0	65	10.4	228	9.2
Private	464	74.0	560	89.6	1024	81.8
**Years since hypertension diagnosis**						
Median, IQR^a^	6.3	8.4	6.6	8.9	6.4	8.8
**Systolic blood pressure (mm Hg)**						
Mean, SD^b^	134.2	18.3	133.4	17.5	133.8	17.9
**Diastolic blood pressure (mm Hg)**						
Mean, SD	86.9	10.0	86.8	9.8	86.8	9.9
**Waist circumference (cm)**						
Mean, SD	93.6	9.8	95.3	10.3	94.4	10.1
**Hip circumference (cm)**						
Mean, SD	98.7	9.7	100.5	10.4	99.6	10.1
**Global dietary requirement score** (0–18, higher score is better diet quality)						
Mean, SD	10.7	1.8	9.8	1.9	10.2	1.9
**Hypertension knowledge** (0–21, higher score is better knowledge)						
Mean, SD	11.8	2.6	12.2	2.2	12.0	2.4
**Perceived social support**						
Median, IQR	4	1.2	4	1.1	4.0	1.1
Low social support (below median)	315	50.2	314	50.2	629	50.2
High social support (above median)	312	49.8	311	49.7	623	49.8
**Controlled blood pressure** (systolic <140 mm Hg and diastolic <90 mm Hg)	342	54.6	354	56.6	696	55.6
**Physical activity**						
≥600 Metabolic equivalents of task	334	53.3	320	51.2	654	52.2
**Body mass index**						
Normal weight (<25 kg/m^2^)	183	29.2	187	29.9	370	29.6
Overweight (25–29 kg/m^2^)	290	46.2	286	45.8	576	46.0
Obese (≥30 kg/m^2^)	154	24.6	152	24.3	306	24.4
**Waist to hip ratio**						
High (≥1)	162	25.8	160	25.6	322	25.7
**Daily salt intake (g)**						
Mean, SD	12.1	3.8	14.6	4.4	13.3	4.3
<5 g	2	0.3	1	0.2	3	0.2
≥5 g	625	99.7	624	99.8	1249	99.8
**Adherence to antihypertensives**						
Good adherence (>6 MMAS^c^)	470	77.9	394	68.3	864	73.2

a Interquartile range.

b Standard Deviation.

c Morisky medication adherence scale.

**Fig. 3:**
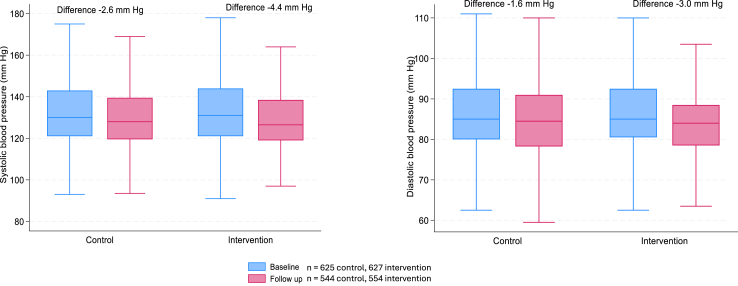
**Change in systolic and diastolic blood pressure from baseline to follow-up**.

The median time from baseline to follow-up data collection was 11.9 (IQR 2.1) months in the intervention arm and 12.4 (IQR 2.6) in the control arm. Baseline and follow up measures of each outcome variable are described in Supplementary Table S5. The mean systolic blood pressure decreased from 133.4 to 130.8 mm Hg (2.6 mm Hg) in the control arm, and from 134.2 to 129.8 mm Hg (4.4 mm Hg) in the intervention arm (Fig. 3). The mean difference in diastolic blood pressure from baseline to follow up was 3.0 mm Hg and 1.6 mm Hg in the intervention and control arm, respectively.

In the primary analysis a higher reduction in systolic blood pressure (1.7 mm Hg, 95% CI −0.1, 3.4) was seen in the intervention group compared to control, but the difference between the groups was not statistically significant (Table 3). The intraclass correlation for systolic blood pressure was very low (<0.001). The mean decrease in diastolic blood pressure was 1.6 mm Hg (95% CI 0.5, 2.6) more in the intervention arm compared to control (Table 3). In the sensitivity analysis (adjusted model), the estimated mean difference in systolic blood pressure was slightly larger, but the mean difference in diastolic blood pressure remained stable.

**Table 3:** Intention-to-treat analysis of intervention effect on the primary and secondary outcomes using mixed model.

Trial outcomes	Primary analysis		Sensitivity analysis^b^	
Primary outcome	Difference (95% CI^a^)	p value	Difference (95% CI)	p value
Systolic blood pressure (mm Hg)	−1.7 (−3.4, 0.1)	0.058	−1.8 (−3.6, −0.1)	0.040
**Secondary outcomes**				
Diastolic blood pressure (mm Hg)	−1.6 (−2.6, −0.5)	0.003	−1.6 (−2.6, −0.5)	0.003
Waist circumference (cm)	2.5 (1.7, 3.2)	<0.001	2.5 (1.7, 3.2)	<0.001
Hip circumference (cm)	1.4 (0.6, 2.3)	<0.001	1.5 (0.6, 2.4)	<0.001
Global dietary requirement score (higher score represents better diet quality)	−1.4 (−1.7, −1.1)	<0.001	−1.4 (−1.7, −1.1)	<0.001
Daily salt intake (g)	−0.7 (−0.9, −0.5)	<0.001	−0.7 (−0.9, −0.5)	<0.001
Hypertension knowledge score (higher score represents better knowledge)	0.7 (0.3, 1.1)	<0.001	0.7 (0.3, 1.1)	<0.001
	**Odds ratio (95% CI)**	**p value**	**Odds ratio (95% CI)**	**p value**
Controlled blood pressure (Reference ≥140/90 mm Hg)	1.5 (1.0, 2.1)	0.040	1.5 (1.0, 2.2)	0.025
High physical activity (Reference <600 MET^c^)	2.2 (1.6, 3.1)	<0.001	2.2 (1.6, 3.1)	<0.001
High body mass index (Reference <25 kg/m^2^)	1.0 (0.5, 2.1)	0.965	1.2 (0.5, 2.7)	0.624
High waist to hip ratio (Reference <1)	1.8 (1.1, 2.9)	0.020	1.7 (1.1, 2.9)	0.029
Good adherence to antihypertensives (Reference <6 MMAS^d^)	0.9 (0.6, 1.5)	0.822	0.9 (0.6, 1.5)	0.838
High perceived social support (Reference < median MSPSS^e^)	1.7 (1.2, 2.4)	0.002	1.7 (1.2, 2.4)	0.002

a Confidence interval.

b Adjusted for age (continuous), gender (male/female), education (years of schooling), marital status (currently married/unmarried) and income (continuous).

c Metabolic equivalents of task.

d Morisky medication adherence scale.

e Multidimensional scale for perceived social support.

The daily dietary salt intake decreased more (0.7 g 95% CI 0.5, 0.9) in the intervention arm compared to control. The proportion with blood pressure control (OR 1.5 95% CI 1.0, 2.1), engaging in adequate physical activity (≥600 METs per week) (OR 2.2, 95% CI 1.6, 3.1) and reporting to receive perceived social support (OR 1.7, 95% CI 1.2, 2.4) were higher in intervention arm compared to control. The change in hypertension knowledge score was higher (0.7, 95% CI 0.3, 1.1) in the intervention arm compared to control. The waist and hip circumference and waist to hip ratio increased more, and global dietary requirement scores decreased more in the intervention group. There was no detectable effect on the body mass index and adherence to antihypertensives. The intervention effects on the secondary outcomes did not change in the adjusted model. The results of the per protocol analysis (Supplementary Table S6) were consistent with the intention-to-treat analysis.

The effects of the intervention on the primary outcome varied slightly in most predefined subgroups but there was no evidence of heterogeneity (Supplementary Figure S1). Exploratory post-hoc subgroup analysis showed no statistically significant difference in the effect of intervention on the primary outcome by baseline blood pressure levels (cutoff at ≥160/100 mm Hg), diabetes status and age group (Supplementary Table S7). The effect of intervention on secondary outcomes by sex are provided in supplementary Table S8. There was heterogeneity by sex in hip circumference with higher effect for males (*P*_interaction_ = 0.007) and hypertension knowledge with higher effect for females (*P*_interaction_ = 0.001).

## Discussion

The trial tested the effectiveness of a comprehensive intervention package on blood pressure, diet quality, adherence to antihypertensives, hypertension knowledge, physical activity, body mass index, waist to hip ratio and perceived social support. CHWs facilitated home visits to reinforce healthy lifestyles and medication adherence and encouraging routine monitoring and audit of blood pressure achieved a modest reduction in blood pressure and daily dietary salt intake and some improvement in hypertension knowledge. Furthermore, compared to the control group the intervention group appeared to achieve greater increase in the proportion with controlled blood pressure, engaged in adequate physical activity, and perceived to receive high social support. However, the intervention seemed to have a negative effect on waist to hip ratio, hip and waist circumference, and diet quality.

Modest intervention effects on blood pressure similar to ours were also reported by community trials from India^31^ and Kenya.^32^ One possible reason for observing a modest effect could be that our intervention didn't improve diet quality, body mass index, or adherence to antihypertensives. Our intervention decreased the daily dietary salt intake, but it was still higher than the WHO recommendation (≤5 g per day). Extending the intervention period might have reduced salt intake further contributing to additional decline in blood pressure. Furthermore, the secular decline in blood pressure from baseline to follow-up in the control arm might have attenuated our ability to detect a significantly higher decline as hypothesised for this trial. Other studies from Nepal,^33^ Pakistan,^34^ India,^35^, ^36^, ^37^ South Asia^13^ and Argentina^38^ have reported significantly greater reduction in blood pressure among individuals with hypertension. The contrasting results is likely because participants in our trial may have had better access to hypertension care and antihypertensives at baseline, leaving less scope for improvement. Also, it is worth noting that the nature of the intervention such as the qualification and training of CHWs, the frequency, duration, content, and approach used during home visits, linkage, and level of support from the health system varied across these studies. Therefore, bigger trials with broader geographical scope are needed to establish effectiveness of multicomponent interventions. Similarly, our post hoc analysis suggested that the intervention may have had a better effect on reducing blood pressure among males compared to females, though the statistical evidence for difference between males and females was weak (Supplementary Table S7 and Supplementary Figure S1). A well powered trial to explore the effectiveness on different subgroups is recommended.

Our study, like other comprehensive interventions^33^^,^^39^ that measured adherence had no effect on adherence to antihypertensives, while a study from South Asia reported significant improvement in MMAS score.^13^ The lack of effect in our study may be because most participants were accustomed to taking antihypertensives (long duration since diagnosis) and had good adherence at baseline. Studies have shown that delay in initiating treatment (antihypertensives) is common among recently diagnosed hypertension patients,^17^^,^^40^ highlighting the importance of providing interventions to newly diagnosed patients.

Similar to our study, others found no effects of comprehensive interventions on weight,^35^^,^^38^^,^^41^ body mass index^37^^,^^39^^,^^41^ and waist to hip ratio.^41^ The possible explanation for not seeing improvements in these anthropometric measurements could be rising population level obesity and particularly in this age group.^4^ Greater reduction in the waist to hip ratio and improvements in diet quality among control participants could explain the subdued intervention effect. Our intervention increased the proportion engaged in adequate physical activity however, this alone is insufficient in reducing weight without improvements in diet quality.^42^ Participants were supported in adopting healthy behaviours including diet but people need resources (financial and infrastructure) to adopt a healthy lifestyle.^43^ Another plausible reason for lack of effect on diet could be subadditivity of intervention effects. The combined effect of a multicomponent intervention is less than the sum of interventions that implement each component alone.^44^ The effect on diet quality may have been underestimated in comparison to trials solely focused on diet modification.

### Strengths and weaknesses of the study

Our study implemented a highly contextualised comprehensive intervention, which was informed by detailed, formative research with potential participants, and used a rigorous cluster randomised controlled trial design to evaluate impact following an analysis plan in the published protocol.^18^ The intervention package included government recommended interventions and focused on problem solving, capacity building, and family participation. We leveraged on the increased acceptance of the CHWs as an integral part of the health system^45^ achieving high fidelity to CHW facilitated home visits. Continuous monitoring of the CHWs ensured that the interventions were relevant and need based. We included adults with controlled and uncontrolled hypertension achieving a good follow-up rate. Our results are free from the influence of regression to the mean as our analysis forced the baseline values to be similar in both the groups.^30^ We implemented a rigorous analysis method, sensitivity analysis, and participants adherence to intervention was high and loss to follow up rates low.

Our trial has some limitations. We used an open-label, non-blinded cluster randomised trial design because one component of our intervention, i.e. the training of public healthcare providers, was at community level. This approach prevented us from recruiting individual participants prior to randomization. The intervention effect might have been underestimated because participants in the control group may have modified their behaviour in response to blood pressure measurements at baseline. Also, public healthcare providers from the control facilities may have improved performance intensifying community awareness activities. To minimise bias in outcome assessment, roles for data collection and intervention were kept separate. The control and intervention clusters were geographically close, thus raising the possibility of contamination. However, none of the control participants had reported interacting with CHWs or seeking care from the intervention public health facilities during follow-up interviews. Our trial was underpowered to detect changes in some of the secondary outcomes. We also had few clusters, but all clusters enrolled at baseline were retained. Our trial may have selection bias as not all eligible adults were screened for hypertension. Those aware of health in general are more likely to participate and thus may limit the generalizability of our findings. The short duration of the intervention meant that there was insufficient time to delve into the underlying causes of uncontrolled hypertension to strategize actions for the sustained behavioural change. Self-reported outcomes such as diet quality, salt intake, and adherence to antihypertensives could bias our study results, although we used validated and recommended instruments. Physical activity measured as METs may be over or underestimated due to misclassification in reporting intensity, duration, and frequency of physical activity. The social support and medication adherence may be complex constructs for a population with low education and could have measurement error. Also, anthropometric measurements using a portable tool may be prone to measurement error.

### Implications

Our trial of a comprehensive intervention showed a modest decrease in blood pressure among hypertension patients with relatively low literacy levels in urban Nepal. Our intervention contains components that are already prioritised by the national and local governments, albeit with little convergence at a community level.^46^ The national government supports districts and municipalities to implement the PEN program through training and logistical support,^47^ however, affordable community strategies for blood-pressure control are lacking. Efforts could be optimised ensuring convergence of programs from different levels of care to accelerate hypertension management.

Programs involving patients and family to self-monitor blood pressure, make lifestyle changes and adhere to medications coupled with health system strengthening are needed. Blood pressure monitoring device was provided to encourage home monitoring of blood pressure, but the high cost may reduce feasibility during intervention-scale up. However, it can be advocated to include the device in the health insurance package. In our trial, the proportion of females was higher even though data collection and home visits were scheduled on weekends and after office hours for facilitating recruitment and retention of men. A probable reason for low male participation could be high male labour migration in Nepal.^48^ Any future intervention should identify innovative strategies to reach males in the community. Our findings can be generalised to similar populations in other parts of South Asia. However, generalising results from cluster randomised trials requires additional information about context, mechanisms, outcomes, and interactions between these, which our planned process evaluation will explore.^49^ Our intervention might perform better among recently diagnosed hypertension patients and in context with high trust in the public health facilities. Improving supply of antihypertensives may help to increase trust in public health facilities creating an enabling environment for effective management of hypertension.^17^

### Conclusion

We found that a comprehensive intervention for hypertension care that is anchored on home visits by trained CHWs for patient support and training of public healthcare providers for routine follow-up is feasible and can control blood pressure in urban Nepal. These approaches have been implemented separately in different contexts and could be scaled up together optimising effects on hypertension control to substantially reduce the societal burden of hypertension and its complications. However, large-scale studies are needed to establish the generalizability of comprehensive interventions that strengthen the health system for better supply of antihypertensives and follow-up care along with community engagement to address barriers to behavioural change.

## Contributors

SB, AS, and ASen conceptualised the study. SB developed the trial methodology with support from ES, AS, and ASen. SB led all aspects of project administration data collection including training, quality assurance with support from AS, and ASen. SB did the formal analysis and drafted the original manuscript including visualisation. ES and ASen checked the underlying data. ES, AS, BPM, BOÅ and ASen reviewed and provided inputs on the trial protocols, methods, and draft manuscript. All authors reviewed and approved the final manuscript.

## Data sharing statement

Data will be available upon reasonable request to the corresponding author.

## Declaration of interests

All authors declare no competing interests.
