# Supplementary material for: Impact of a community health worker led intervention for improved blood pressure control in urban Nepal: an open-label cluster randomised controlled trial
Source: Lancet Reg Health Southeast Asia. 2024 Aug 8;29:100461. doi: 10.1016/j.lansea.2024.100461 (PMC11364134; doi:10.1016/j.lansea.2024.100461)
Supplement: Supplementary Tables [file mmc1.docx]

**Supplementary table 1. Search terms**

| **MEDLINE search terms in PubMed** | | |
| --- | --- | --- |
| Sn | Concepts | Search terms |
| 1 | Hypertension | hypertension [MESH] OR hypertension [Title/Abstract] OR blood pressure [MESH] OR blood pressure [Title/Abstract] |
| 2 | Provider Education | (((((((Education, continuing [MESH]) OR (physician [Title/Abstract])) OR (nurs*[Title/Abstract])) OR (pharmacist [Title/Abstract])) OR (provider [Title/Abstract])) OR (provider [Title/Abstract])) OR (professional [Title/Abstract])) AND ((train*[Title/Abstract]) OR (education [Title/Abstract])) |
| 3 | Provider Team Change/Task Shifting | Patient Care Team [MESH] OR Primary Nursing [MESH] OR physicians role [MESH] OR physicians practice patterns [MESH] OR health services accessibility [MESH] OR task shifting [Title/Abstract] OR team change [Title/Abstract] OR team-based care [Title/Abstract] OR ((pharmacist[Title/Abstract] OR nurs*[Title/Abstract]) AND (care [Title/Abstract] OR treat*[Title/Abstract])) OR collaborative care [Title/Abstract] OR community health workers [MESH] OR lay health worker [Title/Abstract] |
| 4 | Patient Education and Treatment Adherence | Patient Compliance [MESH] OR patient education as topic [MESH] OR patient participation [MESH] OR patient adherence [Title/Abstract] OR medication adherence [MESH] OR self manage* [Title/Abstract] OR lifestyle modification [Title/Abstract] OR (patient [Title/Abstract] AND (behavior therapy [MESH] OR counsel* OR motiv* [Title/Abstract] OR health behavior [MESH] OR behavior control [MESH] OR health education [MESH] OR health promotion [MESH] OR health coaching [Title/Abstract])) |
| 5 | Health Care Provider Feedback and Guideline Adherence | Decision Support Systems, Clinical [MESH] OR Decision Making, Computer Assisted [MESH] OR Reminder Systems [MESH] OR Practice Guideline [MESH] OR Guidelines as topic [MESH] OR Medical Audit [MESH] OR Guideline Adherence [MESH] OR reimbursement, incentive [MESH] OR reimbursement mechanisms [MESH] OR feedback [Title/Abstract] OR guideline adherence [Title/Abstract] OR stepped care [Title/Abstract] |
| 6 | Home Blood Pressure Monitoring/Telemonitoring | ((self [Title/Abstract] OR home [Title/Abstract]) AND monitor* [Title/Abstract]) OR telemedicine [MESH] OR telemonitor* [Title/Abstract] OR telecare [Title/Abstract] OR telehealth [Title/Abstract] OR telehomecare [Title/Abstract] |
| 7 | Patient Reminders | Patient[Title/Abstract] AND (electronic [Title/Abstract] OR text[Title/Abstract] OR adheren* [Title/Abstract] OR complian* [Title/Abstract]) AND (remind* [Title/Abstract] OR alarm* [Title/Abstract] OR message* [Title/Abstract]) |
| 8 |  | 2 OR 3 OR 4 OR 5 OR 6 OR 7 |
| 9 |  | ((((((((LLMIC*[Title/Abstract]) OR ("Low income countr*"[Title/Abstract])) OR ("Lower Middle income countr*"[Title/Abstract])) OR ("low and lower middle income countr*"[Title/Abstract])) OR ("low income and lower middle income countr*"[Title/Abstract])) OR ("Developing countries"[Mesh])) OR ("Developing countr*"[Title/Abstract])) OR ("Afghanistan"[Mesh] OR "Algeria"[Mesh] OR "Angola"[Mesh] OR "Bangladesh"[Mesh] OR "Benin"[Mesh] OR "Bhutan"[Mesh] OR "Bolivia"[Mesh] OR "Burundi"[Mesh] OR "Burkina Faso"[Mesh] OR "Cambodia"[Mesh] OR "Cote d Ivoire"[Mesh] OR "Cameroon"[Mesh] OR "Chad"[Mesh] OR "Comoros "[Mesh] OR "Congo"[Mesh] OR "Cabo Verde"[Mesh] OR "Central African Republic"[Mesh] OR "Djibouti"[Mesh] OR "Egypt"[Mesh] OR "Eritrea"[Mesh] OR "Ethiopia"[Mesh] OR "Eswatini"[Mesh] OR "Gambia"[Mesh] OR "Ghana"[Mesh] OR "Guinea"[Mesh] OR "Guinea-Bissau"[Mesh] OR "Haiti"[Mesh] OR "Honduras"[Mesh] OR "India"[Mesh] OR "Iran"[Mesh] OR "Jordan"[Mesh] OR "Kenya"[Mesh] OR "Lesotho"[Mesh] OR "Liberia"[Mesh] OR "Lebanon"[Mesh] OR "Madagascar"[Mesh] OR "Malawi"[Mesh] OR "Mali"[Mesh] OR "Mauritania"[Mesh] OR "Micronesia"[Mesh] OR "Mongolia"[Mesh] OR "Morocco"[Mesh] OR "Mozambique"[Mesh] OR "Myanmar"[Mesh] OR "Nepal"[Mesh] OR "Nicaragua"[Mesh] OR "Niger"[Mesh] OR "Nigeria"[Mesh] OR "Pakistan"[Mesh] OR "Philippines"[Mesh] OR "Papua New Guinea"[Mesh] OR "Rwanda"[Mesh] OR "Senegal"[Mesh] OR "Somalia"[Mesh] OR "Sudan"[Mesh] OR "Sierra Leone"[Mesh] OR "Syria"[Mesh] OR "South Sudan"[Mesh] OR "Samoa"[Mesh] OR "Sri Lanka"[Mesh] OR "sao Tome and Principe"[Mesh] OR "Tanzania"[Mesh] OR "Togo"[Mesh] OR "Tunisia"[Mesh] OR "Tajikistan"[Mesh] OR "Timor-Leste"[Mesh] OR "Uganda"[Mesh] OR "Ukraine"[Mesh] OR "Uzbekistan"[Mesh] OR "Vanuatu "[Mesh] OR "Vietnam"[Mesh] OR "Yemen"[Mesh] OR "Zambia"[Mesh] OR "Zimbabwe"[Mesh] OR "Democratic People's Republic of Korea"[Mesh])) OR "Kyrgyz Republic"[Title/Abstract] OR "Lao PDR"[Title/Abstract] OR "Solomon Islands"[Title/Abstract] OR "Kiribati"[Title/Abstract]) |
| 10 | Low and lower middle-income countries | 1 AND 8 AND 9 |
| 11 |  | 10 Limited to Humans |
| 12 |  | 11 Limited to Adults |
| 13 |  | 12 Limit to Clinical Trials, All |
| 14 |  | 13 October 2017, and October 2023 |
| **EMBASE Search terms** | | |
| 1 | Search Terms for Hypertension | ‘hypertension‘/exp OR hypertension:ti,ab OR ‘blood pressure’/exp OR ‘blood pressure’:ti,ab |
| 2 | Search Terms for Provider Education | ‘Continuing education’/exp OR ((physician:ti,ab OR nurs*:ti,ab OR pharmacist:ti,ab OR provider:ti,ab OR professional:ti,ab) AND (education:ti,ab OR train*:ti,ab)) |
| 3 | Search Terms for Provider Team Change/Task Shifting | ‘Patient Care Team’:ti,ab OR ‘Primary Nursing’/exp OR ‘physicians role’:ti,ab OR ‘physicians practice patterns’:ti,ab OR ‘health services accessibility’:ti,ab OR ‘task shifting’:ti,ab OR ‘team change’:ti,ab OR ‘team-based care’:ti,ab OR ((pharmacist:ti,ab OR nurs*:ti,ab) AND (care:ti,ab OR treat*:ti,ab)) OR ‘collaborative care’:ti,ab OR ‘community health worker’:ti,ab OR ‘lay health worker’:ti,ab |
| 4 | Search Terms for Patient Education and Treatment Adherence | ‘Patient Compliance’/exp OR ‘patient education’/exp OR ‘patient participation’/exp OR ‘patient adherence’:ti,ab OR ‘medication compliance’/exp OR ‘medication adherence’:ti,ab OR ‘self manage’:ti,ab OR ‘self management’:ti,ab OR ‘self managing’:ti,ab OR ‘lifestyle modification’:ti,ab OR (patient:ti,ab AND (‘behavior therapy’/exp OR counsel*:ti,ab OR motiv*:ti,ab OR ‘health behavior’/exp OR ‘behavior control’/exp OR ‘health education’/exp OR ‘health promotion’/exp OR ‘health coaching’:ti,ab)) |
| 5 | Search Terms for Health Care Provider Feedback and Guideline Adherence | ‘Decision Support System’/exp OR ‘Reminder System’/exp OR ‘Practice Guideline’/exp OR ‘Medical Audit’/exp OR ‘Guideline Adherence’:ti,ab OR reimbursement/exp OR feedback:ti,ab OR ‘guideline adherence’:ti,ab OR ‘stepped care’:ti,ab |
| 6 | Search Terms for Home Blood Pressure Monitoring/Telemonitoring | ((self:ti,ab OR home:ti,ab) AND monitor*:ti,ab) OR telemedicine/exp OR telemonitor*:ti,ab OR telecare:ti,ab OR telehealth:ti,ab OR telehomecare:ti,ab |
| 7 | Search Terms for Patient Reminders | Patient:ti,ab AND (electronic:ti,ab OR text:ti,ab OR adheren*:ti,ab OR complian*:ti,ab) AND (remind*:ti,ab OR alarm*:ti,ab OR message*:ti,ab) |
| 8 |  | 2 OR 3 OR 4 OR 5 OR 6 OR 7 |
| 9 | Low and lower middle-income countries | ('afghanistan'/exp OR 'algeria'/exp OR 'angola'/exp OR 'bangladesh'/exp OR 'burkina faso'/exp OR 'benin'/exp OR 'bhutan'/exp OR 'bolivia'/exp OR 'burundi'/exp OR 'cambodia'/exp OR 'cote d ivoire' OR 'cameroon'/exp OR 'comoros'/exp OR 'congo'/exp OR 'cabo verde'/exp OR 'central african republic'/exp OR 'djibouti'/exp OR 'egypt'/exp OR 'eritrea'/exp OR 'ethiopia'/exp OR 'eswatini'/exp OR 'gambia'/exp OR 'ghana'/exp OR 'guinea'/exp OR 'guinea-bissau'/exp OR 'haiti'/exp OR 'honduras'/exp OR 'india'/exp OR 'iran'/exp OR 'jordan'/exp OR 'kenya'/exp OR 'lebanon'/exp OR 'lesotho'/exp OR 'liberia'/exp OR 'madagascar'/exp OR 'malawi'/exp OR 'mali'/exp OR 'mauritania'/exp OR 'moldova'/exp OR 'micronesia'/exp OR 'mongolia'/exp OR 'morocco'/exp OR 'mozambique'/exp OR 'myanmar'/exp OR 'nepal'/exp OR 'nicaragua'/exp OR 'niger'/exp OR 'nigeria'/exp OR 'pakistan'/exp OR 'papua new guinea'/exp OR 'philippines'/exp OR 'rwanda'/exp OR 'senegal'/exp OR 'sierra leone'/exp OR 'somalia'/exp OR 'sudan'/exp OR 'south sudan'/exp OR 'syria'/exp OR 'somoa'/exp OR 'sri lanka'/exp OR 'tanzania'/exp OR 'sao tome and principe'/exp OR 'togo'/exp OR 'tunisia'/exp OR 'timor-leste'/exp OR 'tajikistan'/exp OR 'uganda'/exp OR 'ukraine'/exp OR 'vanuatu'/exp OR 'uzbekistan'/exp OR 'vietnam'/exp OR 'yemen'/exp OR 'zambia'/exp OR 'zimbabwe'/exp OR 'democratic peoples republic of korea' OR 'west bank and gaza' OR 'kiribati'/exp OR 'kyrgyz republic'/exp OR 'lao pdr' OR 'solomon islands'/exp) OR 'llmic*':ti,ab,kw OR 'low income country':ti,ab,kw OR 'lower middle income country':ti,ab,kw OR 'lower middle income country'/exp OR 'low income country'/exp OR 'low and lower middle income countr*':ti,ab,kw OR 'low income and lower middle income countr*':ti,ab,kw OR 'developing country'/exp OR 'developing countries':ti,ab,kw |
| 10 |  | 8 AND 1 |
| 10 |  | 11 Limited to Humans |
| 11 |  | 12 Limited to Adults |
| 12 |  | 13 Limit to Clinical Trials, All |

**Supplementary table 2: Inclusion and exclusion criteria**

| **Criterion** | **Include** | **Exclude** |
| --- | --- | --- |
| Publication type | Peer reviewed articles | abstracts, reports, briefs, conference proceedings |
| Study Type | Randomised controlled trials | Trial protocols, articles describing development of intervention, reporting on baseline findings, feasibility, and cost-effectiveness analysis |
| Intervention | Interventions targeting one or more levels of barriers (individual, provider, social and health system) compared to usual care | Testing effect of treatment without addressing any levels of barriers to treatment |
| Trial duration | At least 6 months | Follow up period of less than 6 months |
| Outcomes | Net change in systolic and diastolic blood pressure | Health outcomes not related to hypertension |
| Study population | Adults with hypertension | General population or other conditions (e.g. obesity, diabetes, pregnant women) |
| Location | Low and lower middle-income countries | Middle and high-income countries |
| Year of Publication | October 2018- December 2023 |  |

**Supplementary table 3. Summary of findings from 13 hypertension trials testing interventions ≥ 6 months (14 articles) from low and lower middle-income countries (3 articles from 2018 review by Mills and colleges(1) and 11 from updating their review for articles between October 2017 - December 2023)**

| **Author, Year of Publication, Country** | **Group** | **Baseline Participant Characteristics** | | | | | **Study Characteristics** | | | | | **Results** | | |
| --- | --- | --- | --- | --- | --- | --- | --- | --- | --- | --- | --- | --- | --- | --- |
|  |  | **N** | **Men, %** | **Mean Age, years** | **BP Medication, %** | **SBP/DBP,**  **mmHg** | **Study Design** | **Clusters (if applicable); Participants** | **Duration** | **Key Components of Implementation Strategy** | **Description of Usual Care** | **SBP mean difference (95%CI)** | **DBP mean difference (95%CI)** |  |
| Khan et al. 2019, Pakistan(2) | Intervention | 574 | 50.5 | 45.7 | - | 161.3/103.6 | C | 26 private clinics  Physician and paramedics  ≥ 25 years patients with  uncontrolled hypertension | 9 months | Usual care plus trained physicians titrating medication and trained paramedics educating on LM. Send text message and/or call to remind for monthly follow-up | Basic training and resources for patient screening | -12.6 mm Hg (-24.6, -0.7) | -7.6 mm Hg  (-14.6, -0.6) |  |
|  | Control | 564 | 47.5 | 44.6 | - | 1161.4/03.5 |  |  |  |  |  |  |  |  |
| Jafar et al. 2020, South Asia (Pakistan, Sri Lanka and Bangladesh)(3) | Intervention | 1330 | 34.1 | 58.5 | 100 | 146.7/89.1 | C | 30 community (10 per country)  ≥40 years patients with uncontrolled or treated hypertension | 24 months | Home visits by government CHWs HMBP, and LM, MA, and referral for care coordination in the public sector.  Training of the physicians on treatment guidelines. | Usual care | -5.2 mm Hg (-7.1, -3.2) | -1.6 mm Hg (-3.9, -1.7) |  |
|  | Control | 1315 | 37.3 | 59.0 | 100 | 144.7/87.8 |  |  |  |  |  |  |  |  |
| Khetan et al. 2019, India(4) | Intervention | 743 | 63.5 | 62.1 | 100 | 145.9/83.7 | C | 12 clusters; 35-70 years adults with at least 1 CVD risk factors (hypertension, diabetes, and smoking) | 18 months | Home visits by CHWs for LM, MA and encourage follow up care with physicians. | Handout explaining the risk factors of CVDs after screening | −8.9 mm Hg (-14.4, -3.5) | -2.1 mm Hg (-4.5, 0.3) |  |
|  | Control | 689 | 63.5 | 62.4 | 100 | 138.7/80.6 |  |  |  |  |  |  |  |  |
| Malik et al. 2022, Pakistan(5) | Intervention | 40 | 52.1 | --- | --- | 145.9/95.1 | P | Treated, hypertensive and diabetic patients | 6 months | Pharmacist-led counseling on hypertension and diabetes, LM, HMBP. Physician consultation as needed. | Usual care | Not mean difference from baseline Intervention:130.1 mm HG (±6.9)  Control: 145.5 mm Hg (±6.7) | Not mean difference from baseline Intervention: 88.8 mm Hg (±5.4)  Control: 97.0 mm Hg (±6.9) |  |
|  | Control | 40 | 57.5 | --- | --- | 142.2/95.0 |  |  |  |  |  |  |  |  |
| Suseela et al. 2022, India(6) | Intervention | 968 | 40 | 56.8 | 50 | 147.7/89.1 | C | 20 slums  ≥18 years  Diagnosed, hypertensive patients | 6 months | Home visits and peer group meetings by women self-help group members engaging participants and family members to discuss on LM, HMBP, MA and regular follow up visit and set behavioural goals and discuss problems in achieving them. | Usual care | -4.1 mm Hg (-6.1, -2.2) | -1.6 mm Hg (- 2.6, -0.5) |  |
|  | Control | 984 | 39 | 55.7 | 56 | 145.5/88.2 |  |  |  |  |  |  |  |  |
| Prabhakaran, 2019, India(7) | Intervention | 1159 | 57.3 | 55.8 | --- | 152.5/88.8 | C | 40 PHC  ≥30 years patients diagnosed with hypertension | 12 months | Physicians used EDS for treatment including medicine titration and follow up. Nurses used EDS to tailor LM counselling. Reminders sent for follow-up and MA. | Physician trained on clinical management tablets provided to the facility for data collection | -0.31 mm Hg (−3.91, 3.29] | --- |  |
|  | Control | 1131 | 53.1 | 54.4 | --- | 157.0/93.3 |  |  |  |  |  |  |  |  |
| Neupane et al. 2018, Nepal(8) | Intervention | 255 | 47 | 50.1 | 35 | 142.7/91.8 | C | 14 communities  25-65 years Diagnosed hypertension. | 12 months | Home visits by female community health volunteer for BP monitoring and personalized counselling LM and MA. | Usual care | - 4·9 mm Hg (-7·8, 2·0) | -2·6 mm Hg (-4·6, 0·7) |  |
|  | Control | 180 | 36 | 50.3 | 34 | 144.2/93.1 |  |  |  |  |  |  |  |  |
| Thapa et al. 2023, Nepal(9) | Intervention | 227 | 46.7 | 50.2 | 34.4 | 142.9/91.9 | C | 14 communities  25-65 years  Diagnosed hypertension. | 60 months | Home visits by female community health volunteer for BP monitoring and personalized counselling LM and MA. | Usual care | 4.1 mm Hg (2.2,6.1) | 1.6 mm Hg (0.5, 2.6) |  |
|  | Control | 168 | 35.1 | 49.9 | 35.1 | 143.1/92.8 |  |  |  |  |  |  |  |  |
| Tavakoly et al. 2020, Iran(10) | Intervention | 119 | 21.8 | 53.8 | 100 | 145.6/91.5 | P | Physicians and ≥ 18 years  Uncontrolled hypertensive patients from PCC | 6 months | Physician applied Health Literacy in Practice model (HLPM) for patient counselling. HLPM emphasizes understanding patient’s concerns, health literacy and self-management abilities to address barrier. | Usual care | Intervention: -21.4 mm Hg (±6.1)  Control: 2.3 mm Hg (±1.03) | Intervention: 13.0 mm Hg (±3.5)  Control: 2.4 mm Hg (±0.7) |  |
|  | Control | 121 | 19.8 | 54.1 | 100 | 146.1/89.5 |  |  |  |  |  |  |  |  |
| Narayana et al. 2019, India(11) | Intervention | 105 | 75.2 | 43.7 | 87.6 | 147.6/89.3 | P | ≥ 18 years  Patients with hypertension and comorbidity | 6 months | Pharmacist led counseling on BP monitoring, LM and MA | Usual care | Not mean difference from baseline Intervervention:138 mm Hg (±10.5)  Control:146 mm Hg (± 8.2) | Not mean difference from baseline  Intervention: 82.3mm Hg (± 7.2)  Control: 87.2 mm Hg (± 5.4) |  |
|  | Control | 105 | 78.1 | 43.9 | 85.7 | 149.0/88.2 |  |  |  |  |  |  |  |  |
| Vedanthan, 2019, Kenya(12) | Intervention 1 | 469 | 44 | 54.3 | 100 | 161.3/89.7 | C | 24 cluster  >20 years uncontrolled hypertension | 18 months | Intervention 1: CHW used smartphones to facilitated EDS for tailored behavioural intervention | Usual care | −13.1 mm Hg smartphone  -8.4 paper based.  −9.7 in usual care. | 1.5 mm Hg smartphone  0.4 paper based.  0.1 in usual care. |  |
|  | Intervention 2 | 500 | 44 | 53.7 | 100 | 158.3/90.4 |  |  |  | Intervention 2: CHW used paper-based information to provide tailored behavioural intervention |  |  |  |  |
|  | Control | 491 | 37 | 54.6 | 100 | 158.4/89.0 |  |  |  |  |  |  |  |  |
| Saleem et al. 2015, Pakistan(13) | Intervention | 193 | 64.8 | 39 | 100 | 144.5/90.5 | P | Treated, hypertensive patients | 9 months | Pharmacist-led sessions for MA and LM; educational materials | Usual care | Not mean difference from baseline Intervervention:137.5 mm Hg (±17.2)  Control:143.9 mm Hg (± 19.4) | Not mean difference from baseline  Intervention:84.6 mm Hg (± 9.9)  Control: 90.1 mm Hg (± 10.5) |  |
|  | Control | 192 | 72.9 |  | 100 | 144.1/90.9 |  |  |  |  |  |  |  |  |
| Jafar et al. 2009, Pakistan (14) | Intervention 1 | 335 | 41.2 | 55.3 | 35.5 | 153.3/92.9 | C | 12 Low- to middle-income, communities; Hypertensive patients | 24 months | Physicians training program for guideline concordant care and patient communication | Usual care | -10.8 mm Hg (-12.8, -8.9) in intervention 2 compared to usual care | -1.6 mm Hg (- 2.6, -0.5)  in intervention 2 compared to usual care |  |
|  | Intervention 2 | 348 | 38.2 | 52.7 | 39.7 | 151.8/93.7 |  |  |  | CHW-led home visits for LM and MA; social support |  |  |  |  |
|  | Intervention 3 | 332 | 33.7 | 54 | 35.2 | 148.3/91.1 |  |  |  | Intervention 1 and 2 combined |  |  |  |  |
| Anchala et al. 2015, India(15) | Intervention | 845 | 49.4 | 35-64 | --- | 151.0/89.4 | C | 16 PCCs; Hypertensive patients | 12 months | Computer-based decision support system for BP management | Educational materials | -6.59 mm Hg (-12.18, -1.42) | -2.83 mm Hg (-5.78, 0.13) |  |
|  | Control | 793 | 51.6 | 35-64 | --- | 148.0/88.3 |  |  |  |  |  |  |  |  |
| Abbreviations: PCC – primary care center, CHW – community health worker, BP – blood pressure, MA – medication adherence, LM – lifestyle modification, HBPM – home blood pressure monitoring, CVD – cardiovascular disease, EDS – electronic decision support, P – parallel study design, C – cluster-randomised study design | | | | | | | | | | | | | | |
|  |  |  |  |  |  |  |  |  |  |  |  |  |  |  |

**Supplementary table 4: Associations with loss to follow-up by trial arm**

| **Characteristic** | **Intervention** | | | | | | **Control** | | | | | |
| --- | --- | --- | --- | --- | --- | --- | --- | --- | --- | --- | --- | --- |
|  | **Retained** | | | **Loss to follow-up** | | | **Retained** | | | **Loss to follow-up** | | |
|  | **N** | **Freq.** | **%** | **N** | **Freq.** | **%** | **N** | **Freq.** | **%** | **N** | **Freq.** | **%** |
| **Age** |  |  |  |  |  |  |  |  |  |  |  |  |
| Mean, SD* | 554 | 57.8 | 11.9 | 73 | 57.7 | 13.9 | 544 | 57.3 | 11.9 | 81 | 57.2 | 13.3 |
| 21- 44 years | 84 | 73 | 86.9 |  | 11 | 13.1 | 100 | 84 | 84.0 |  | 16 | 16.0 |
| 45- 59 years | 270 | 236 | 87.4 |  | 34 | 12.6 | 257 | 224 | 87.2 |  | 33 | 12.8 |
| ≥ 60 years | 273 | 245 | 89.7 |  | 28 | 10.3 | 268 | 236 | 88.1 |  | 32 | 11.9 |
| **Education** |  |  |  |  |  |  |  |  |  |  |  |  |
| Illiterate | 212 | 185 | 87.3 |  | 27 | 12.7 | 201 | 173 | 86.1 |  | 28 | 13.9 |
| Primary (0-4 grade) | 213 | 192 | 90.1 |  | 21 | 9.9 | 245 | 214 | 87.4 |  | 31 | 12.6 |
| Secondary (5-10 grade) | 118 | 101 | 85.6 |  | 17 | 14.4 | 102 | 90 | 88.2 |  | 12 | 11.8 |
| High school and above | 84 | 76 | 90.5 |  | 8 | 9.5 | 77 | 67 | 87.0 |  | 10 | 13.0 |
| **Family Size** |  |  |  |  |  |  |  |  |  |  |  |  |
| Mean, SD |  | 4.6 | 2.0 |  | 4.4 | 2.1 |  | 4.5 | 1.8 |  | 4.3 | 2.1 |
| **Sex** |  |  |  |  |  |  |  |  |  |  |  |  |
| Male | 259 | 228 | 88.0 |  | 31 | 12.0 | 241 | 207 | 85.9 |  | 34 | 14.1 |
| Female | 368 | 326 | 88.6 |  | 42 | 11.4 | 384 | 377 | 87.7 |  | 47 | 12.2 |
| **Marital status** |  |  |  |  |  |  |  |  |  |  |  |  |
| Married | 531 | 471 | 88.7 |  | 60 | 11.3 | 519 | 451 | 86.9 |  | 68 | 13.1 |
| Unmarried | 96 | 83 | 86.5 |  | 13 | 13.5 | 106 | 93 | 87.7 |  | 13 | 12.3 |
| **Ethnicity** |  |  |  |  |  |  |  |  |  |  |  |  |
| Brahmin/Chettri | 326 | 293 | 89.9 |  | 33 | 10.1 | 348 | 302 | 86.8 |  | 46 | 13.2 |
| Newar | 139 | 131 | 94.2 |  | 8 | 5.8 | 99 | 89 | 89.9 |  | 10 | 10.1 |
| Tamang/ Rai/ Sherpa/ Magar/ Gurung | 118 | 94 | 79.7 |  | 24 | 20.3 | 142 | 119 | 83.8 |  | 23 | 16.2 |
| Dalits | 44 | 36 | 81.8 |  | 8 | 18.2 | 36 | 34 | 94.4 |  | 2 | 5.6 |
| **Occupation** |  |  |  |  |  |  |  |  |  |  |  |  |
| Unemployed | 376 | 331 | 88.0 |  | 45 | 12.0 | 315 | 272 | 86.4 |  | 43 | 13.6 |
| Retired | 52 | 47 | 90.4 |  | 5 | 9.6 | 80 | 74 | 92.5 |  | 6 | 7.5 |
| Paid employment | 54 | 47 | 87.0 |  | 7 | 13.0 | 52 | 43 | 82.7 |  | 9 | 17.3 |
| Self employed | 145 | 129 | 89.0 |  | 16 | 11.0 | 178 | 155 | 87.1 |  | 23 | 12.9 |
| **Per-capita annual income (USD**)** |  |  |  |  |  |  |  |  |  |  |  |  |
| Mean, SD |  | 980.2 | 1127.3 |  | 976.1 | 1490.1 |  | 1245.4 | 1567.2 |  | 1453.0 | 2854.4 |
| *Income tertiles* |  |  |  |  |  |  |  |  |  |  |  |  |
| Low (<577 USD) | 270 | 235 | 87.0 |  | 35 | 13.0 | 162 | 127 | 78.4 |  | 35 | 21.6 |
| Medium (578-1150 USD) | 182 | 159 | 87.4 |  | 23 | 12.6 | 221 | 199 | 90.1 |  | 22 | 9.9 |
| High (> 1150 USD) | 175 | 160 | 91.4 |  | 15 | 8.6 | 242 | 218 | 90.1 |  | 24 | 9.9 |
| **Standard alcoholic drinks** |  |  |  |  |  |  |  |  |  |  |  |  |
| Non-drinkers | 476 | 421 | 88.5 |  | 55 | 11.5 | 527 | 457 | 86.7 |  | 70 | 13.3 |
| <3 drinks per week | 46 | 42 | 91.3 |  | 4 | 8.7 | 26 | 24 | 92.3 |  | 2 | 7.7 |
| ≥3 drinks per week | 105 | 91 | 86.7 |  | 14 | 13.3 | 72 | 63 | 87.5 |  | 9 | 12.5 |
| **Current tobacco users** | 131 | 111 | 84.7 |  | 20 | 15.3 | 130 | 119 | 91.5 |  | 11 | 8.5 |
| **Diabetic** | 140 | 124 | 88.6 |  | 16 | 11.4 | 130 | 121 | 93.1 |  | 9 | 6.9 |
| **Number of antihypertensives prescribed** |  |  |  |  |  |  |  |  |  |  |  |  |
| 0 | 30 | 25 | 83.3 |  | 5 | 16.7 | 52 | 43 | 82.7 |  | 9 | 17.3 |
| 1 | 307 | 273 | 88.9 |  | 34 | 11.1 | 291 | 251 | 86.3 |  | 40 | 13.7 |
| 2 | 247 | 220 | 89.7 |  | 27 | 10.9 | 251 | 223 | 88.8 |  | 28 | 11.2 |
| 3-4 | 43 | 36 | 83.7 |  | 7 | 16.3 | 31 | 27 | 87.1 |  | 4 | 12.9 |
| **Type of health facility for seeking hypertension care** |  |  |  |  |  |  |  |  |  |  |  |  |
| Public | 163 | 149 | 91.4 |  | 14 | 8.9 | 65 | 58 | 89.2 |  | 7 | 10.8 |
| Private | 464 | 405 | 87.3 |  | 59 | 12.7 | 560 | 486 | 86.8 |  | 74 | 13.2 |
| **Years since hypertension diagnosis** |  |  |  |  |  |  |  |  |  |  |  |  |
| Mean, SD |  | 8.3 | 7.1 |  | 9.0 | 9.5 |  | 8.2 | 7.0 |  | 8.8 | 8.6 |
| **Systolic blood pressure (mm Hg)** |  |  |  |  |  |  |  |  |  |  |  |  |
| Mean, SD |  | 134.0 | 17.9 |  | 135.4 | 20.8 |  | 132.7 | 17.1 |  | 137.6 | 19.7 |
| **Diastolic blood pressure (mm Hg)** |  |  |  |  |  |  |  |  |  |  |  |  |
| Mean, SD |  | 86.9 | 10.1 |  | 86.2 | 9.0 |  | 86.5 | 9.6 |  | 88.9 | 10.8 |
| **Waist Circumference (cm)** |  |  |  |  |  |  |  |  |  |  |  |  |
| Mean, SD |  | 93.4 | 9.7 |  | 95.0 | 10.8 |  | 95.5 | 10.4 |  | 93.9 | 9.5 |
| **Hip Circumference (cm)** |  |  |  |  |  |  |  |  |  |  |  |  |
| Mean, SD |  | 98.5 | 9.6 |  | 99.7 | 10.4 |  | 100.6 | 10.5 |  | 99.6 | 10.1 |
| **Global dietary requirement score** (0 to 18, higher score better diet quality) |  |  |  |  |  |  |  |  |  |  |  |  |
| Mean, SD | 554 | 10.7 | 1.8 | 73 | 10.6 | 1.6 | 544 | 9.8 | 1.8 | 81 | 9.6 | 1.7 |
| **Hypertension knowledge** (0-21, higher score better knowledge) |  |  |  |  |  |  |  |  |  |  |  |  |
| Mean, SD | 554 | 12.2 | 2.5 | 73 | 11.8 | 2.6 | 544 | 12.2 | 2.2 | 81 | 12.3 | 2.1 |
| **Perceived social support** |  |  |  |  |  |  |  |  |  |  |  |  |
| Mean, SD |  | 4.0 | 0.9 |  | 3.9 | 0.9 |  | 4.1 | 0.7 |  | 4.0 | 0.7 |
| Low social support (below median) | 315 | 274 | 87.0 |  | 41 | 13.0 | 314 | 272 | 86.6 |  | 42 | 13.4 |
| High social support (above median) | 312 | 280 | 89.7 |  | 32 | 103 | 311 | 272 | 87.5 |  | 39 | 12.5 |
| **Controlled blood pressure** (systolic <140 mm Hg and diastolic <90 mm Hg) | 342 | 305 | 89.2 |  | 37 | 10.8 | 354 | 317 | 89.5 |  | 37 | 10.5 |
| **Physical activity** |  |  |  |  |  |  |  |  |  |  |  |  |
| ≥600 Metabolic equivalents of task | 334 | 292 | 87.4 |  | 42 | 12.6 | 320 | 275 | 85.9 |  | 45 | 14.1 |
| **Body mass index** |  |  |  |  |  |  |  |  |  |  |  |  |
| Normal weight (<25 kg/m^2^) | 183 | 160 | 87.4 |  | 23 | 12.6 | 187 | 156 | 83.4 |  | 31 | 16.6 |
| Overweight (25-29 kg/m^2^) | 290 | 264 | 91.0 |  | 26 | 9.0 | 286 | 250 | 87.4 |  | 36 | 12.6 |
| Obese (≥30 kg/m^2^) | 154 | 130 | 84.4 |  | 24 | 15.6 | 152 | 138 | 90.8 |  | 14 | 9.2 |
| **Waist to hip ratio** |  |  |  |  |  |  |  |  |  |  |  |  |
| High (≥1) | 162 | 141 | 87.0 |  | 21 | 13.0 | 160 | 143 | 89.4 |  | 17 | 10.6 |
| **Daily salt intake (grams)** |  |  |  |  |  |  |  |  |  |  |  |  |
| Mean, SD |  | 11.7 | 3.7 |  | 14.6 | 4.2 |  | 14.2 | 4.2 |  | 17.2 | 4.3 |
| <5 grams | 2 | 2 | 100.0 |  | 0 | 0.0 | 1 | 1 | 100.0 |  | 0 | 0.0 |
| ≥ 5 grams | 625 | 552 | 88.3 |  | 73 | 11.7 | 624 | 543 | 87.0 |  | 81 | 13.0 |
| **Adherence to antihypertensives** |  |  |  |  |  |  |  |  |  |  |  |  |
| Good adherence (>6 MMAS) | 470 | 410 | 87.2 |  | 60 | 12.8 | 394 | 343 | 87.1 |  | 51 | 12.9 |
| *Standard deviation, **USD=United States Dollars (Exchange rate 1USD=130 Nepali rupees), ¤Morisky medication adherence scale | | | | | | | | | | | | |

**Supplementary table 5. Outcomes at baseline and follow-up**

|  | **Baseline** | | | | **Follow-up** | | | |
| --- | --- | --- | --- | --- | --- | --- | --- | --- |
|  | **Intervention**  **N = 627** | | **Control**  **N= 625** | | **Intervention**  **N=554** | | **Control**  **N =544** | |
| **PRIMARY OUTCOME** | **Mean** | **SD*** | **Mean** | **SD** | **Mean** | **SD** | **Mean** | **SD** |
| **Systolic blood pressure (mm Hg)** | 134.2 | 18.3 | 133.4 | 17.5 | 129.8 | 16.2 | 130.8 | 18.0 |
| **SECONDARY OUTCOMES** |  |  |  |  |  |  |  |  |
| **Diastolic blood pressure (mm Hg)** | 86.9 | 10.0 | 86.8 | 9.8 | 83.9 | 9.8 | 85.2 | 10.4 |
| **Waist circumference (cm)** | 93.6 | 9.8 | 95.3 | 10.3 | 93.5 | 9.9 | 92.8 | 10.6 |
| **Hip circumference (cm)** | 98.7 | 9.7 | 100.5 | 10.4 | 97.7 | 9.0 | 98.1 | 9.9 |
| **Global dietary requirement score**  (higher score is better diet quality) | 10.7 | 1.8 | 9.8 | 1.9 | 10.8 | 1.5 | 11.3 | 1.8 |
| **Hypertension knowledge score**  (higher score is better knowledge) | 11.8 | 2.6 | 12.2 | 2.2 | 14.1 | 2.5 | 13.7 | 2.9 |
|  | **Freq** | **%** | **Freq** | **%** | **Freq** | **%** | **Freq** | **%** |
| **MSPSS #** |  |  |  |  |  |  |  |  |
| Mean, SD | 4.0 | 0.9 | 4.1 | 0.7 | 4.1 | 1.0 | 4.2 | 0.7 |
| Median, IQR** | 4 | 1.2 | 4 | 1.1 | 4.7 | 1.3 | 4 | 1.3 |
| Low MSPSS (below median) | 315 | 50.2 | 314 | 50.2 | 240 | 55.2 | 300 | 55.1 |
| High MSPSS (above median) | 312 | 49.8 | 311 | 49.7 | 300 | 43.3 | 244 | 44.9 |
| **Controlled BP** | 342 | 54.6 | 354 | 56.6 | 376 | 67.9 | 344 | 63.2 |
| **Physical activity** |  |  |  |  |  |  |  |  |
| ≥600 MET*** | 342 | 54.6 | 320 | 56.6 | 376 | 67.9 | 279 | 51.3 |
| **Body mass index** |  |  |  |  |  |  |  |  |
| Normal weight (<25 kg/m^2^) | 183 | 29.2 | 187 | 29.9 | 160 | 28.9 | 158 | 29.0 |
| Overweight (≥25 kg/m^2^) | 444 | 70.8 | 438 | 70.1 | 394 | 71.1 | 386 | 71.0 |
| **Daily salt intake in grams** |  |  |  |  |  |  |  |  |
| Mean, SD | 12.1 | 3.8 | 14.6 | 4.4 | 10.9 | 4.4 | 14.1 | 4.4 |
| < 5 grams | 2 | 0.3 | 1 | 0.2 | 14 | 2.5 | 3 | 0.6 |
| ≥ 5 grams | 625 | 99.7 | 624 | 99.8 | 540 | 97.5 | 541 | 99.4 |
| **Fruits and vegetable intake** |  |  |  |  |  |  |  |  |
| <400 grams per day | 553 | 88.2 | 514 | 82.2 | 516 | 93.1 | 414 | 76.1 |
| **Waist to hip ratio** |  |  |  |  |  |  |  |  |
| High ≥ 1 | 162 | 25.8 | 160 | 25.6 | 159 | 28.7 | 125 | 23.0 |
| **Adherence to antihypertensives** | N= 603 | | N= 577 | | N= 525 | | N= 512 | |
| Poor adherence (≤ 6 MMAS¤) | 133 | 22.1 | 183 | 31.7 | 65 | 12.4 | 89 | 17.4 |
| Good adherence (>6 MMAS) | 470 | 77.9 | 394 | 68.3 | 460 | 87.6 | 423 | 82.6 |

*Standard deviation, **interquartile range, ***Metabolic equivalents of task (MET), # Multidimensional scale for perceived social support, ¤Morisky medication adherence scale

**Supplementary table 6: Per protocol analysis of intervention effect on the primary and secondary outcomes using mixed model (excluding 163 participants with no outcome measurements at follow-up and/or <5 home visits).**

| **TRIAL OUTCOMES** | **Per protocol** | | **Adjusted**** | |
| --- | --- | --- | --- | --- |
| **PRIMARY OUTCOME** | **Difference (95% CI*)** | **p value** | **Difference (95% CI)** | **p value** |
| Systolic blood pressure (mm Hg) | -1.6 (-3.4, 0.1) | 0.072 | -1.7 (-3.5, -0.0) | 0.052 |
| **SECONDARY OUTCOMES** |  |  |  |  |
| Diastolic blood pressure (mm Hg) | -1.6 (-2.6, 0.5) | 0.003 | -1.6 (-2.6, 0.6) | 0.003 |
| Waist circumference (cms) | 2.5 (1.7, 3.3) | <0.001 | 2.5 (1.7, 3.3) | <0.001 |
| Hip circumference (cms) | 1.5 (0.6, 2.3) | 0.001 | 1.5 (0.6, 2.4) | 0.001 |
| Global dietary requirement score (higher score is better diet quality) | -1.4 (-1.7, 1.1) | <0.001 | -1.4 (-1.7, 1.1) | <0.001 |
| Daily salt intake(grams) | -0.7 (-1.0, -0.5) | <0.001 | -0.7 (-1.0, -0.5) | <0.001 |
| Hypertension knowledge score (higher score is better knowledge) | 0.7 (0.4, 1.2) | 0.001 | 0.8 (0.4, 1.2) | 0.001 |
|  | **Odds ratio (95% CI)** | **p value** | **Odds ratio (95% CI)** | **p value** |
| Controlled blood pressure (Reference ≥140/90 mm Hg) | 1.5 (1.0, 2.2) | 0.048 | 1.5 (1.0, 2.2) | 0.031 |
| High physical activity  (Reference <600 MET***) | 2.2 (1.6, 3.1) | <0.001 | 2.3 (1.7, 3.2) | <0.001 |
| High body mass index  (Reference <25 kg/m^2^) | 1.0 (0.5, 2.1) | 0.977 | 1.2 (0.6, 2.6) | 0.640 |
| High waist to hip ratio  (Reference <1) | 1.8 (1.1, 3.0) | 0.015 | 1.8 (1.1, 3.0) | 0.019 |
| Good adherence to antihypertensives (Reference <6 MMAS¤) | 1.0 (0.6, 1.5) | 0.921 | 1.0 (0.6, 1.6) | 0.942 |
| High perceived social support (Reference < median MSPSS#) | 1.7 (1.2, 2.4) | 0.003 | 1.7 (1.2, 2.4) | 0.002 |

*Confidence interval, **Adjusted for age (continues), gender (male/ female), education (years of schooling), marital status (currently married/unmarried) and income (continuous), *** Metabolic equivalents of task; ¤ Morisky medication adherence scale, # Multidimensional scale for perceived social support,

**Supplementary table 7: Intervention effect on the primary outcome (systolic blood pressure) by subgroup using mixed model**

| **Subgroups** | **Baseline** | | **Follow-up** | | **difference (95% CI*)** | **p value** | **P value for interaction** |
| --- | --- | --- | --- | --- | --- | --- | --- |
|  | **Intervention**  **N =627** | **Control**  **N=625** | **Intervention**  **N =554** | **Control**  **N= 544** |  |  |  |
| **Sex** |  |  |  |  |  |  |  |
| Female | 368 | 384 | 326 | 337 | -0.4 (-2.6, 1.9) | 0.736 | 0.064 |
| Male | 259 | 241 | 228 | 207 | -3.8 (-6.5, -1.0) | 0.008 |  |
| **Income (median income cutoff)** |  |  |  |  |  |  |  |
| Low income | 336 | 230 | 295 | 189 | -1.7 (-4.1, 0.6) | 0.151 | 0.760 |
| High income | 291 | 395 | 259 | 355 | -2.3 (-5.0, 0.4) | 0.095 |  |
| **Body mass index** |  |  |  |  |  |  |  |
| Normal weight (<25 kg/m^2^) | 183 | 187 | 160 | 156 | -3.4 (-6.6, 0.2) | 0.042 | 0.228 |
| Overweight (≥25 kg/m^2^) | 444 | 438 | 394 | 388 | -1.0 (-3.1, 1.0) | 0.341 |  |
| **Waist to hip ratio** |  |  |  |  |  |  |  |
| Low (<1) | 465 | 465 | 413 | 401 | -1.3 (-3.4, 0.7) | 0.199 | 0.515 |
| High (≥1) | 162 | 160 | 141 | 143 | -2.7 (-6.1, 0.8) | 0.130 |  |
| **Post hoc exploratory** | | | | | | | |
| **Blood pressure status** |  |  |  |  |  |  |  |
| Poorly controlled (≥160/100mm Hg) | 285 | 271 | 249 | 227 | -4.7 (-8.9, 0.6) | 0.026 | 0.110 |
| Well controlled (<160/100mm Hg) | 342 | 354 | 305 | 317 | -1.1 (-2.8, 0.7) | 0.229 |  |
| **Age** |  |  |  |  |  |  |  |
| < 60 years | 354 | 357 | 309 | 308 | -1.1 (-3.4, 1.2) | 0.349 | 0.454 |
| ≥ 60 years | 273 | 268 | 245 | 236 | -2.5 (-5.1, 0.2) | 0.068 |  |
| **Diabetes status** |  |  |  |  |  |  |  |
| Non-diabetic | 487 | 495 | 430 | 432 | -2.0 (-4.0, 0.0) | 0.051 | 0.557 |
| Diabetic | 140 | 130 | 124 | 121 | -0.7 (-4.4, 3.0) | 0.705 |  |
| *Confidence interval | | | | | | | |

**Supplementary table 8: Intervention effect on secondary outcomes by sex using mixed model**

| **TRIAL OUTCOMES** | **Male** | | **Female** | | **p value for interaction** |
| --- | --- | --- | --- | --- | --- |
| **SECONDARY OUTCOMES** | **Difference**  **(95% CI*)** | **p value** | **Difference**  **(95% CI)** | **p value** |  |
| Diastolic blood pressure (mm Hg) | -2.5 (-4.2, -0.8) | 0.005 | -0.9 (-2.1, -0.5) | 0.200 | 0.093 |
| Waist circumference (cm) | 2.3 (1.2, 3.5) | <0.001 | 2.5 (1.5, 3.5) | <0.001 | 0.897 |
| Hip circumference (cm) | 2.8 (1.5, 4.0) | <0.001 | 0.5 (-0.6, 1.7) | 0.374 | 0.007 |
| Global dietary requirement score (higher score is better diet quality) | -1.5 (-1.9, -1.1) | <0.001 | -1.3 (-1.6, -0.9) | <0.001 | 0.217 |
| Daily salt intake (grams) | -0.6 (-0.9, -0.2) | 0.001 | -0.8 (-1.1, -0.5) | <0.001 | 0.489 |
| Hypertension knowledge score (higher score is better knowledge) | 0.1 (-0.4, 0.7) | 0.696 | 1.0 (0.5, 1.5) | <0.001 | 0.001 |
|  | **Odds ratio**  **(95% CI)** | **p value** | **Odds ratio**  **(95% CI)** | **p value** |  |
| Controlled blood pressure (Reference ≥140/90 mm Hg) | 1.7 (1.0, 3.1) | 0.067 | 1.3 (0.8, 2.0) | 0.327 | 0.301 |
| High physical activity  (Reference <600 MET**) | 1.8 (1.1, 2.9) | 0.029 | 2.6 (1.7, 4.0) | <0.001 | 0.199 |
| High body mass index  (Reference <25 kg/m^2^) | 1.7 (0.5, 5.3) | 0.389 | 1.4 (0.4, 5.0) | 0.590 | 0.310 |
| High waist to hip ratio  (Reference <1) | 1.2 (0.6, 2.2) | 0.020 | 2.8 (1.4, 5.8) | 0.006 | 0.074 |
| Good adherence to antihypertensives (Reference <6 MMAS¤) | 0.8 (0.4, 1.8) | 0.669 | 1.1 (0.6, 2.0) | 0.746 | 0.413 |
| High perceived social support (Reference < median MSPSS#) | 1.4 (0.9, 2.4) | 0.174 | 1.9 (1.3, 2.9) | 0.002 | 0.317 |
| *Confidence interval **Metabolic equivalents of task; ¤Morisky medication adherence scale; #Multidimensional scale for perceived social support | | | | | |

**References**

1. Mills KT, Obst KM, Shen W, Molina S, Zhang HJ, He H, et al. Comparative Effectiveness of Implementation Strategies for Blood Pressure Control in Hypertensive Patients: A Systematic Review and Meta-analysis. Ann Intern Med. 2018;168(2):110-20.

2. Khan MA, Khan N, Walley JD, Khan SE, Hicks J, Sheikh FI, et al. Enhanced hypertension care through private clinics in Pakistan: A cluster randomised trial. BJGP Open. 2019;3(1).

3. Jafar TH, Gandhi M, de Silva HA, Jehan I, Naheed A, Finkelstein EA, et al. A Community-Based Intervention for Managing Hypertension in Rural South Asia. N Engl J Med. 2020;382(8):717-26.

4. Khetan A, Zullo M, Rani A, Gupta R, Purushothaman R, Bajaj NS, et al. Effect of a Community Health Worker-Based Approach to Integrated Cardiovascular Risk Factor Control in India: A Cluster Randomized Controlled Trial. Glob Heart. 2019;14(4):355-65.

5. Malik M, Hussain A, Aslam U, Hashmi A, Vaismoradi M, Hayat K, et al. Effectiveness of Community Pharmacy Diabetes and Hypertension Care Program: An Unexplored Opportunity for Community Pharmacists in Pakistan. Frontiers in Pharmacology. 2022;13.

6. P Suseela R, Ambika RB, Mohandas S, Menon JC, Numpelil M, K Vasudevan B, et al. Effectiveness of a community-based education and peer support led by women's self-help groups in improving the control of hypertension in urban slums of Kerala, India: a cluster randomised controlled pragmatic trial. BMJ Global Health. 2022;7(11).

7. Prabhakaran D, Jha D, Prieto-Merino D, Roy A, Singh K, Ajay VS, et al. Effectiveness of an mHealth-Based Electronic Decision Support System for Integrated Management of Chronic Conditions in Primary Care: The mWellcare Cluster-Randomized Controlled Trial. Circulation. 2019;139(3):380-91.

8. Neupane D, McLachlan CS, Mishra SR, Olsen MH, Perry HB, Karki A, et al. Effectiveness of a lifestyle intervention led by female community health volunteers versus usual care in blood pressure reduction (COBIN): an open-label, cluster-randomised trial. Lancet Glob Health. 2018;6(1):e66-e73.

9. Thapa R, Zengin A, Neupane D, Mishra SR, Koirala S, Kallestrup P, et al. Sustainability of a 12-month lifestyle intervention delivered by community health workers in reducing blood pressure in Nepal: 5-year follow-up of the COBIN open-label, cluster randomised trial. The Lancet Global Health. 2023;11(7):e1086-e95.

10. Tavakoly Sany SB BF, Ferns G, et al. Communication skills training for physicians improves health literacy and medical outcomes among patients with hypertension: a randomized controlled trial. BMC Health Services Research. 2020;20(1):60-.

11. Narayana G, Goruntla N, Mallela V, Nayakanti D. Effect of pharmacist directed counselling services on knowledge, attitude, and practice (KAP) and blood pressure control in hypertensive patients: A randomized control trial. International Journal of Pharmaceutical Sciences and Research. 2019;10(11):5109-16.

12. Vedanthan R, Kamano JH, DeLong AK, Naanyu V, Binanay CA, Bloomfield GS, et al. Community Health Workers Improve Linkage to Hypertension Care in Western Kenya. J Am Coll Cardiol. 2019;74(15):1897-906.

13. Saleem F, Hassali MA, Shafie AA, Ul Haq N, Farooqui M, Aljadhay H, et al. Pharmacist intervention in improving hypertension-related knowledge, treatment medication adherence and health-related quality of life: a non-clinical randomized controlled trial. Health expectations : an international journal of public participation in health care and health policy. 2015;18(5):1270-81.

14. Jafar TH, Hatcher J, Poulter N, Islam M, Hashmi S, Qadri Z, et al. Community-based interventions to promote blood pressure control in a developing country: a cluster randomized trial. Ann Intern Med. 2009;151(9):593-601.

15. Anchala R, Kaptoge S, Pant H, Di Angelantonio E, Franco OH, Prabhakaran D. Evaluation of effectiveness and cost-effectiveness of a clinical decision support system in managing hypertension in resource constrained primary health care settings: results from a cluster randomized trial. J Am Heart Assoc. 2015;4(1):e001213.
