# Supplementary material for: Impact of a community health worker led intervention for improved blood pressure control in urban Nepal: an open-label cluster randomised controlled trial
Source: Lancet Reg Health Southeast Asia. 2024 Aug 8;29:100461. doi: 10.1016/j.lansea.2024.100461 (PMC11364134; doi:10.1016/j.lansea.2024.100461)
Supplement: Supplementary Figure S1 [file mmc2.pdf]

Supplementary figure 1. Change in systolic blood pressure by subgroups according to characteristics of participants at baseline

Subgroups	Number of Participants: Intervention	Number of Participants: Control	Mean Difference (95% CI)	P-value for interaction
Overall	627	625	-1.7 (-3.4, 0.1)	
Sex				
Male	259	241	-3.8 (-6.5, -1.0)	
Female	368	384	-0.4 (-2.6, 1.9)	0.064
Income *				
Low	336	230	-1.7 (-4.1, 0.6)	
High	291	395	-2.3 (-5.0, 0.4)	0.760
Body mass index				
<25 kg/m²	183	187	-1.0 (-3.1, 1.0)	
≥25 kg/m²	444	438	-3.4 (-6.6, 0.2)	0.228
Waist to hip ratio ¤				
High	162	160	-2.7 (-6.1, 0.8)	
Low	465	465	-1.3 (-3.4, 0.7)	0.515
Blood pressure $				
Well controlled	342	354	-1.1 (-2.8, 0.7)	
Poorly controlled	285	271	-4.7 (-8.9, 0.6)	0.110

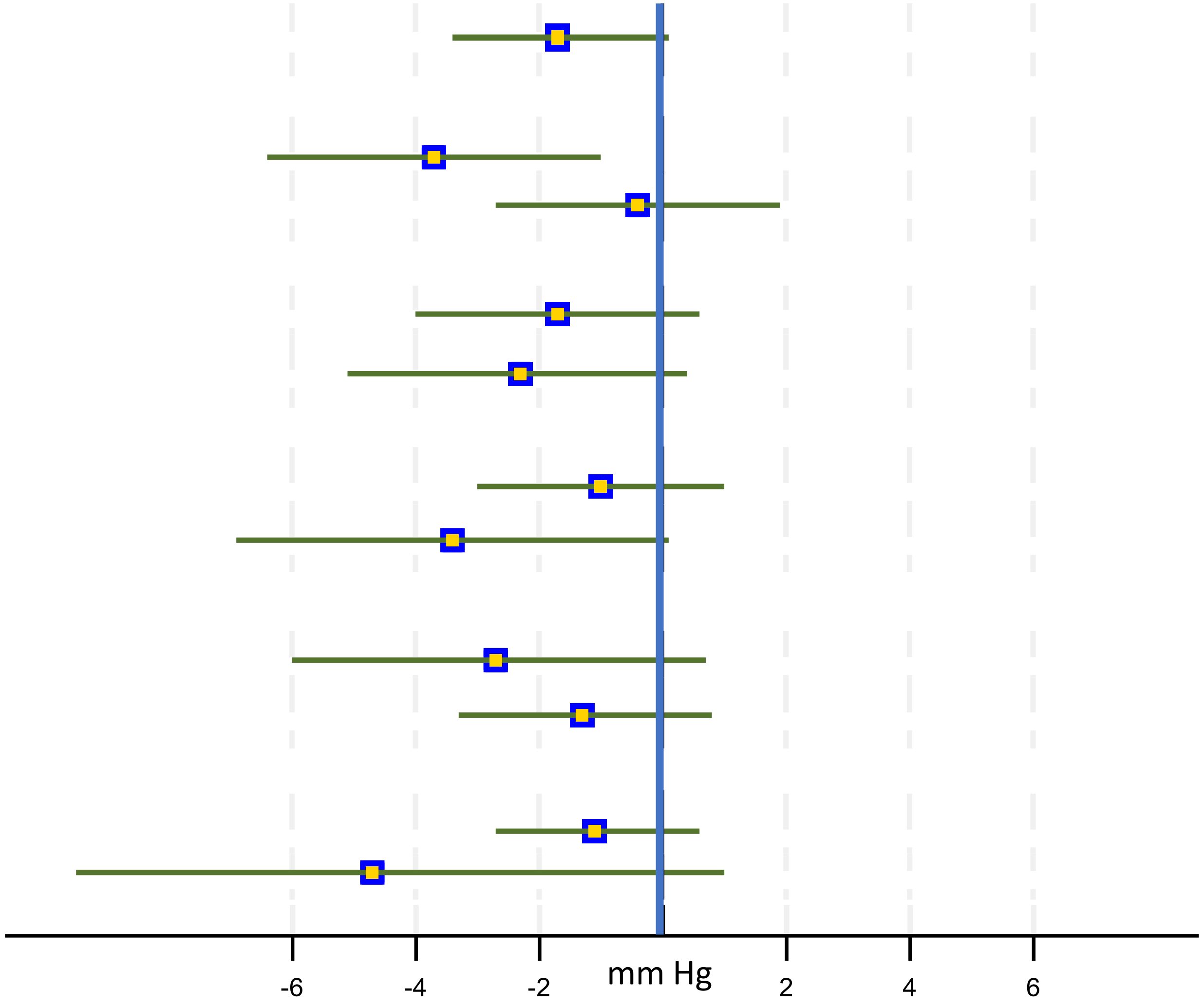

*Per-capita income (median cutoff income)
¤waist to hip ratio (<1 and ≥1)
$Well controlled systolic <160 mm Hg and diastolic <100 mm Hg and poorly controlled systolic ≥160 mm Hg and diastolic ≥100 mm Hg
